# Characterization of the Single Stranded DNA Binding Protein SsbB Encoded in the Gonoccocal Genetic Island

**DOI:** 10.1371/journal.pone.0035285

**Published:** 2012-04-19

**Authors:** Samta Jain, Maria Zweig, Eveline Peeters, Katja Siewering, Kathleen T. Hackett, Joseph P. Dillard, Chris van der Does

**Affiliations:** 1 Department of Microbiology, Groningen Biomolecular Sciences and Biotechnology Institute, University of Groningen, Groningen, The Netherlands; 2 Research Group of Microbiology, Department of Sciences and Bio-engineering Sciences, Vrije Universiteit Brussel, Brussels, Belgium; 3 Medical Microbiology and Immunology, University of Wisconsin, Madison, Wisconsin, United States of America; 4 Department of Ecophysiology, Max-Planck-Institute for Terrestrial Microbiology, Marburg, Germany; University of South Florida College of Medicine, United States of America

## Abstract

**Background:**

Most strains of *Neisseria gonorrhoeae* carry a Gonococcal Genetic Island which encodes a type IV secretion system involved in the secretion of ssDNA. We characterize the GGI-encoded ssDNA binding protein, SsbB. Close homologs of SsbB are located within a conserved genetic cluster found in genetic islands of different proteobacteria. This cluster encodes DNA-processing enzymes such as the ParA and ParB partitioning proteins, the TopB topoisomerase, and four conserved hypothetical proteins. The SsbB homologs found in these clusters form a family separated from other ssDNA binding proteins.

**Methodology/Principal Findings:**

In contrast to most other SSBs, SsbB did not complement the *Escherichia coli ssb* deletion mutant. Purified SsbB forms a stable tetramer. Electrophoretic mobility shift assays and fluorescence titration assays, as well as atomic force microscopy demonstrate that SsbB binds ssDNA specifically with high affinity. SsbB binds single-stranded DNA with minimal binding frames for one or two SsbB tetramers of 15 and 70 nucleotides. The binding mode was independent of increasing Mg^2+^ or NaCl concentrations. No role of SsbB in ssDNA secretion or DNA uptake could be identified, but SsbB strongly stimulated Topoisomerase I activity.

**Conclusions/Significance:**

We propose that these novel SsbBs play an unknown role in the maintenance of genetic islands.

## Introduction

Single stranded DNA binding proteins (SSBs) are highly conserved, essential proteins found in all kingdoms of life. These proteins bind to single-stranded DNA (ssDNA) with high affinity and low sequence specificity. They play a crucial role in processes involved in DNA metabolism like DNA repair, replication and recombination by modulating the functions of many DNA processing enzymes either by controlling the accessibility to ssDNA or via protein–protein interactions [1]. Several bacteria contain, next to the main SSB, a second SSB. The second SSB can either be found on the chromosome or is located on a plasmid. The presence of a second chromosomal paralog is often related to natural competence [2], [3], [4]. Furthermore, nearly all conjugative plasmids also encode an SSB homologue. The exact function of these SSBs is still unclear; they seem not to be necessary for conjugal transfer of the plasmids, but might be involved in plasmid stability [5]. Remarkably, the SSB homologue VirE2 encoded on the *Agrobacterium tumefaciens* Ti plasmid is essential for infection of the host plant cells [6], [7]. VirE2 is transported via the Type IV secretion system (T4SS) on the Ti plasmid independent of the ssDNA [8]. In the recipient cell, it probably functions as a molecular motor facilitating the import of the Ti ssDNA [9], [10].

The *Escherichia coli* SSB protein has been studied in most detail, and was shown to form a stable homotetramer. The N-terminal domain of each monomer contains an oligonucleotide/oligosaccharide binding (OB) fold [11] that is involved in ssDNA binding and in formation of the tetramer. Many SSBs also contain a disordered acidic C-terminus which is essential for interactions with other proteins [1], [12], [13]. Some bacteria and archaea form homodimeric SSBs and in eukaryotes, heterotrimeric SSBs are found [1]. However, almost all known eubacterial and mitochondrial SSBs function as homotetramers.

The interaction of SSBs with ssDNA has been studied in detail. Depending on the conditions, different binding modes have been observed for tetrameric SSBs. At high protein to DNA ratios, low salt concentrations (<10 mM NaCl) and low Mg^2+^ concentrations (<3 mM), the *E. coli* SSB tetramer binds to ssDNA in the highly cooperative (SSB)_35_ mode. In this mode, SSB binding occludes approximately 35 nucleotides and shows high intratetramer cooperativity. However, at higher salt (>0.2 M NaCl) or Mg^2+^ (>3 mM) concentrations, ssDNA binding occludes approximately 65 nucleotides [14], [15]. This binding mode showed only low cooperativity and was called the (SSB)_65_ mode. Both modes can be interchanged in solution and the physiological relevance of the two binding modes *in vivo* is not clear. The crystal structure of SSB from *E. coli* has been determined in the apo-form and with two (dC)_35_ oligonucleotides bound [15]. Each (dC)_35_ oligonucleotide was bound to two OB folds. The ssDNA is bound in a groove in which both nucleic acid backbone and bases interact with the protein. It has been proposed that in the (SSB)_65_ mode, the ssDNA wraps completely around the SSB tetramer. The cooperative binding of SSB has been visualized by electron microscopy for *E. coli* SSB [16], VirE2 [9], [17], [18], [19] and with Atomic Force Microscopy (AFM) [20] for *E. coli* SSB, the bacteriophage T4 gene 32 protein (gp32) and the yeast Replication Protein A [21].

*N. gonorrhoeae* is a highly naturally competent organism [22], [23]. It encodes a chromosomal SSB that showed DNA binding properties comparable to *E. coli* SSB [24] and a SSB (SsbB) that is encoded within a 57 kb horizontally acquired genetic island called the Gonococcal Genetic Island (GGI). This GGI is found in 85% of the clinical isolates [25]. Approximately half of the GGI encodes a T4SS which is involved in the secretion of ssDNA directly into the medium [26]. The secreted DNA is rapidly taken up by the highly active competence system of Neisseria species and incorporated in the genome. The presence of the T4SS in the GGI increases the transfer rate of chromosomal markers approximately 500 fold [25]. The function of the other half of the GGI is currently unknown. It contains mostly hypothetical proteins, but also putative DNA processing proteins like partitioning proteins *parA* and *parB*, single stranded DNA binding protein *ssbB*, DNA topoisomerase *topB*, DNA helicase *yea* and DNA methylases *ydg* and *ydhA* [26].

The role of the SsbB encoded within this region has not been characterized. We show that SsbB is part of conserved homologous cluster, found in several other proteobacteria. We analyze here the physiological role of this protein belonging to a novel class of SSBs and characterize its function biochemically.

## Materials and Methods

Poly(dT) was purchased from SigmaAldrich. Polynucleotide concentrations are given per nucleotide for poly(dT), and for the complete oligonucleotide for oligonucleotides of determined length dT_n_. Oligonucleotide concentrations were determined spectrophotometrically using an absorption coefficient of 8600 M^−1^ cm^−1^ at 260 nm. Protein concentrations were determined spectrophotometrically at 280 nm using the absorption coefficients calculated from amino acid composition. These concentrations were confirmed by a colorimetric assay using the Bradford reagent from Fermentas.

### Bioinformatics

The synteny of SsbB homologs was determined using Absynte (http://archaea.u-psud.fr/absynte) [27]. Homologs of *N. gonorrhoeae* SsbB and SSB sequences representing the different SSB clusters, based on a recently described set of SSB proteins [28] were retrieved from GenBank (http://www.ncbi.nlm.nih.gov). The resulting SSB dataset included 83 sequences (See Table S1). All sequences were trimmed to contain only the OB-fold domain. Sequences were aligned with MUSCLE (default settings) and then manually refined in MEGA5. MEGA5 was used for Neighboring joining analysis with 500 bootstrap replicates [60].

### Bacterial strains and plasmids

*E. coli* strains were grown in Luria-Bertani (LB) medium at 37°C with the appropriate antibiotics; ampicillin (100 µg/ml), erythromycin (500 µg/ml) or chloramphenicol (34 µg/ml). *N. gonorrhoeae* strains were grown on GCB plates containing Kellogg's supplements at 37°C under 5% CO_2_ [29] or in GCBL liquid medium (15 gr protease peptone, 34 gr K_2_HPO_4_,1 gr KH_2_PO_4_ and 1 gr NaCl in 1 l water) containing 0.042% NaHCO_3_ and Kellogg's supplements or in Graver-Wade medium [30] supplemented with Kellogg's supplements and 0.042% NaHCO_3_. When necessary, chloramphenicol and/or erythromycin were used at 10 µg/ml.

### Construction of plasmids and strains

The strains, plasmids and primers used in this study and their construction are listed in Tables S2, S3 and S4.

### Transcriptional Mapping

*N. gonorrhoeae* strains were grown in GCBL liquid medium containing 0.042% NaHCO_3_ and Kellogg's supplements until OD_600_∼0,6 was reached. Total RNA of 1 ml culture was isolated using the peqGOLD TriFast® reagent (peqLab). To remove contaminating DNA, total RNA was treated with 1 unit RNase-free DNaseI (Fermentas) for 30 min at 37°C. RNA was quantified spectrophotometrically, and quality assessed by agarose gel electrophoresis. The MuLV transcriptase and the random hexamer primer of the first strand cDNA synthesis kit (Fermentas) were used to generate cDNA. A control of cDNA synthesis was performed without MuLV transcriptase. Transcripts were mapped using the following primers: for *yaf-ssbB*, 705R-GGI and 767F-GGI, for *ssb-topB*, 703R-GGI and 702F-GGI, for *topB-yeh*, 708R-GGI and 709F-GGI, for *yeh-yegB*, 701R-GGI and 499F-GGI and for *yegA-yef*, 498R-GGI and 726F-GGI (see Table S4).

### Quantitative PCR

Transcript levels of *ssbB*, *topB*, *traI* and *traD* and the reference gene *secY* were determined for RNA isolated from piliated (EP006) and non piliated (SJ001) *N. gonorrhoeae* strains by quantitative Real-Time PCR (qRT-PCR). Oligonucleotide primers were designed using clone manager 9 professional edition (Sci-Ed Software). The primers used were as follows: for *ssbB*, 766R-GGI and 767F-GGI, for *topB*, 769F-GGI and 770R-GGI, for *traI*, 474R-GGI and 475F-GGI, for *traD*, 472R-GGI and 473F-GGI and for *secY*, 697 and 698 (See Table S4). cDNA was isolated as described above. qRT-PCRs were performed using the SYBR Green/ROX qPCR Master Mix (Fermentas) in a 7300 Real Time PCR System of Applied Biosystems. Reaction mixtures were prepared in a 25 µl volume and run in triplicate for each gene. *N. gonorrhoeae* strain MS11 chromosomal DNA was used to establish the primer efficiency. Six biological replicates were performed. Results were depicted as the level of transcript compared with the *secY* gene (2∧−ΔCt).

### Complementation of *E. coli* SSB

To determine if SsbB could substitute for *E. coli* SSB, we attempted to replace pRDP146 with pKH114 in *E. coli* SSB mutant RDP268. pKH114 carries *ermC* and the GGI *ssb* in place of *tet* and the *E. coli ssb* that are on pRPZ146 [31]. RDP268 carries the *aphA* gene in place of *ssb* on the chromosome and is unable to grow unless complemented with an *ssb* gene [31]. pRDP146 is capable of complementing the mutation as is a similar plasmid carrying the *E. coli* F-plasmid single-stranded binding protein gene *ssf*. Electroporation was used to introduce pKH114 into RDP268, and transformants were selected on LB agar plates containing erythromycin. Fifty Erm^R^ colonies were replica plated to plates containing tetracycline or erythromycin. All fifty transformants grew on both selective media. Plasmid screening by the method of Kado and Liu [32] demonstrated that the Erm^R^ Tet^R^ colonies carried both plasmids, pKH114 and pRDP146. Since these two plasmids carry the same origin of replication, they should be incompatible, and growth without selection for the antibiotic resistance markers would allow for loss of one plasmid if it were not essential for growth [31]. To determine if pRDP146 could be lost, two transformants were grown overnight in Luria broth with erythromycin, but without tetracycline. Dilutions of the culture were plated on LB agar containing erythromycin. 856 Erm^R^ colonies were replica-plated to LB agar containing tetracycline. All Erm^R^ colonies maintained Tet^R^, suggesting that pRDP146 was required for growth and that the GGI SSB could not substitute for *E. coli* SSB in the SSB mutant RDP268.

### Expression and purification of SsbB

SsbB proteins were overexpressed in *E. coli* strain C43 (DE3). Cells were grown in 1 L of Luria-Broth medium at 37°C to an OD_600_ of 0.5 and induced with 0.5 mM isopropyl β-D-1-thiogalactopyranoside (IPTG). After 3 hrs the cells were harvested by centrifugation, resuspended in 30 ml of buffer A (50 mM NaPO_4_, 300 mM NaCl, pH 8.0) and stored at −80°C. Before purification, frozen cell pellets were thawed on ice. After thawing, the solution was supplemented with 1 tablet of Protease Inhibitor Cocktail (Roche) and 1 mgr of DNase I (Roche), and the cells were disrupted 3 times in a high-pressure Cell Disrupter (Constant Cell Disruption Systems) at 2.300 bar. Cell debris was removed by centrifugation at 10.000 rpm in an F10-6x500y rotor (FiberLite) and the supernatant was filtered through a 0.45 µm filter. Purifications were performed on an AKTA-Purifier system (GE Healthcare). His-tagged SsbB was purified by loading the clarified supernatant on a 1 ml Hi-Trap Chelating column (GE Healthcare) preloaded with 0.1 M NiSO_4_ and equilibrated in buffer A. The column was washed with 10 column volumes of buffer A and bound proteins were eluted with a linear gradient of buffer A supplemented with 400 mM imidazol. Peak fractions containing SsbB were pooled and diluted with two volumes of buffer B (10 mM Tris-HCl pH 8.0, 10 mM NaCl). This sample was loaded on a Hi-Trap Q column (GE Healthcare) equilibrated with buffer B, and the protein was eluted with a linear gradient up to 1 M NaCl in buffer B. Fractions containing SsbB were concentrated to 2 ml using Amicon Ultra – 10 K Concentrators (Millipore). Finally these fractions were loaded on a Superdex SD200 gelfiltration column (GE Healthcare), equilibrated witha buffer C containing 150 mM NaCl and 20 mM Tris-HCl pH 8.0. Fractions containing tetrameric SsbB were pooled, and frozen in liquid N_2_ until further usage. OneSTrEP-tagged SsbB was purified by loading the clarified supernatant on a Strep-tactin Sepharose column (IBAGO) equilibrated with buffer D (150 mM NaCl, 1 mM EDTA, 100 mM Tris-HCl pH 8.0). SsbB was eluted with buffer D containing 2.5 mM desthiobiotin. Peak fractions containing SsbB were pooled and diluted with two volumes of buffer B and purified over Hi-Trap Q and Superdex SD200 columns as described above. Native SsbB was purified over a Hi-Trap Q column, as described above. Peak fractions were loaded on a 5 ml Hi-Trap Desalting column (GE Healthcare) equilibrated with buffer E (50 mM NaCl, 1 mM EDTA, 1 mM TCEP, 20 mM Tris-HCl, pH 8.0), fractions containing SsbB were collected. Desalted SsbB fractions were loaded on the DNA-cellulose column (Amersham Bioscience) equilibrated in buffer E and eluted over night at 4°C with buffer E containing 1 M NaCl. Finally SsbB was further purified on a Superdex SD200.

### Polyacrylamide gel electrophoresis mobility shift assays

Most of the ssDNA binding reactions were performed in SBA buffer (10 mM NaOH, 2 mM EDTA, titrated to pH 7.5 with Boric acid) which was when indicated supplemented with 10 mM MgCl_2_ and/or 20, 200 or 500 mM NaCl. Alternatively, experiments were performed in 20 mM Tris/HCl or 25 mM Tris/Acetate pH 7.5. To determine the ssDNA binding affinity of SsbB, a ^32^P 5′-labeled dT_90_ primer was used. 2 nM of the primer was mixed with increasing concentrations of (SsbB)_4_ [0–5000 nM]. To determine the stoichiometry of binding of SsbB, 5′ Cy3 labeled labeled dT_n_ primers were used. 8 nM of dT_35_ or dT_75_ primers were mixed with increasing concentrations of (SsbB)_4_ [0–64 nM]. Both the reactions using radiolabelled and fluorescently labeled primers were incubated at 4°C for 15 min after which the reaction was mixed with 5× gel loading solution (0.25% bromphenol blue, 40% sucrose). The aliquots were analyzed by electrophoresis on 7.5% native polyacrylamide gels using a buffer system consisting of the SBA buffer supplemented with the same MgCl_2_ concentration as used in the binding reaction. The fluorescently labeled primers were visualized on a LAS-4000 imager (Fujifilm). To determine the minimal binding frame of one SsbB tetramer, 1 µM (SsbB)_4_ was incubated with 5 µM of unlabeled dT_n_ oligonucleotide. To determine the minimal binding frame of two SsbB tetramers, 1 µM (SsbB)_4_ was incubated with 0.25 µM of unlabeled dT_n_ oligonucleotide. A similar incubation and separation protocol as described above was used, except that the bands corresponding to the SsbB protein were visualized by G-250 BioSafe Coomassie Brilliant Blue staining.

### Fluorescence titrations

Titrations were performed on a temperature-controlled PC1 spectrofluorometer (ISS Inc) with a cooled photomultiplier. The excitation wavelength was set to 285 nm and the emission wavelength to 340 nm. The slit widths for the excitation and the emission beam were set to 1 and 2 nm respectively. Experiments were performed at 8°C in a buffer containing 20 mM Tris pH 7.5 and 1 mM dithiothreithol. When applicable, 20, 200 or 500 mM NaCl or 10 mM MgCl_2_ was added. Samples were allowed to equilibrate for 90 s between measurements.

### Atomic force microscopy

For AFM measurements, 20 µl binding reactions were prepared in a buffer containing 20 mM Tris pH 7.5, 150 mM NaCl and 150 µM SpdCl_3_. Each reaction mixture contained 50 ng M13 ssDNA (New England Biolabs), and different concentrations of native SsbB. Samples were incubated at 37°C during 10 mins to allow complex formation, after which 10 µl was deposited on freshly cleaved mica. This was incubated during 1 min at room temperature to allow adsorption of the nucleoprotein complexes. Subsequently, the mica surface was rinsed with 100 µl 0.02% uranyl acetate, which stabilizes adsorbed SSB-ssDNA complexes [21]. The mica was rinsed with deionized ultrapure water several times and excess water was blotted off with adsorbing paper. Finally, the mica surface was blown dry in a stream of filtered air. A Nanoscope IIIa microscope (Digital Instruments/Veeco) was operated in the tapping mode, in air. Nanoprobe SPM tips, type RTESP7 (Veeco) were used for imaging of 512×512 pixel images. These tips have a 115–135 µM cantilever, a nominal spring constant of 50 N/m and resonance frequencies in the range from 244 to 295 kHz. Nanoscope 6.11r1 software (Digital Instruments/Veeco) was used to flatten the images and to create zoomed 3D surface plots.

### DNA secretion assays

*N. gonorrhoeae* strains MS11, ND500 and SJ038 were grown overnight on GCB agar plates at 37°C under 5% CO_2_ and inoculated in 3 ml of defined medium (Graver-Wade medium) [30] supplemented with Kellogg's supplements and 0.042% NaHCO_3_ [33]. These cultures were grown while shaking for 1.5 hrs at 37°C under 5% CO_2_ and then diluted to an OD_600_∼0.2. To remove DNA derived from the initial starting culture, the cultures were diluted to OD_600_∼0.1 and growth was continued for 2 hrs. After three dilutions, samples were collected directly after the dilution and after 2 hrs. At these times also the OD_600_ was determined. Cells were directly removed by centrifugation for 5 mins in a table top centrifuge at 14.000 rpm. Supernatants were assayed for the amount of DNA using PicoGreen (Invitrogen). The amount of secreted DNA was calculated by comparison to a DNA standard curve. The amount of secreted DNA was expressed as amount of µg secreted DNA/ΔOD_600_. In all assays *N. gonorrhoeae* ND500 (MS11:ΔGGI) was included as a background.

### Isolation of secreted fraction

To analyze the secreted fraction of *N. gonorrhoeae*, a 250 ml culture of SJ023 was grown to OD_600_ of 0.5 in GCBL medium. Cells were then harvested by centrifugation at 8000 rpm for 10 mins. The medium supernatant was filtered through a 0.2 µm filter to remove the cell debris. The supernatant was centrifuged at 40.000 rpm in a Ti45 rotor for 1 hour at 4°C to obtain the pellet containing the blebs. The pellet was resuspended in 250 µl 2× sample buffer (SB) with 0.5 M Tris-HCl pH 6.8, 10% (w/v) SDS, 0.1% (w/v) bromophenol blue, 20% glycerol and 10 mM DTT. After removal of the pellet, the supernatant fraction was concentrated 100 fold by trichloroacetic acidprecipitation. At higher concentrations the pellets could not be fully resuspended anymore. The harvested cell pellet was resuspended into 20 ml of buffer A (50 mM Tris-HCl pH 7.5) and disrupted using a French press at 15 psi. Cell debris was removed by centrifugation at 6000 rpm for 10 mins and 40 µl supernatant was dissolved with 40 µl of 2× SB and 20 µl was loaded on the gel. Alternatively, either the cytoplasmic supernatant or the medium supernatant obtained from 120 ml culture of SJ023 were applied to a Strep-tactin Sepharose column (IBA) equilibrated with buffer D. Bound proteins were eluted with buffer D containing 2.5 mM desthiobiotin, separated on a 15% SDS-PAGE gel and analyzed by Coomassie staining and Western blotting using an Strep-Tactin AP conjugate antibody (IBA).

### Western Blotting

15% polyacrylamide SDS-PAGE gels were run for all the analyses with SsbB. Western blotting was performed by electroblotting the gels on PVDF membranes and incubating with 1∶4000 dilution of Strep-Tactin AP conjugate antibody (IBA). The chemiluminescence signal was obtained using the CDP-star substrate (Roche) on a LAS-4000 imager (Fujifilm).

### Co-culture assay for DNA uptake and transformation

The assay was performed as described previously [26]. *N. gonorrhoeae* strains EP006 or EP030 which have an erythromycin marker inserted within the *recA* gene to ensure one directional transfer was used as a donor, while *N. gonorrhoeae* strains EP015, EP029 and SJ038 which contain a chromosomal chloramphenicol marker were used as acceptor strains. Shortly, piliated *N. gonorrhoeae* strains were grown overnight on GCB agar plates at 37°C under 5% CO_2_ and transferred to 3 ml of GCBL medium supplemented with Kellogg's supplements and 0.042% NaHCO_3_. Cultures were grown for 2.5 hrs at 37°C with shaking under 5% CO_2_. 1 ml of both donor and recipient cultures were centrifuged and pellets were resuspended in 3 ml of GCBL. 0.5 ml of donor and recipient cells were mixed in 3 ml of GCBL, and growth was continued. After 5 hrs, serial dilutions were spread on selective media containing erythromycin and chloramphenicol. Transfer frequencies were calculated as CFU of transformants per CFU of donor. To study the effect of SsbB on DNA transformation, a similar assay was performed after addition of 50 µl of 2 mg/ml purified OneSTrEP-tagged SsbB directly after mixing the strains and again after 2.5 hrs.

### Topoisomerase DNA relaxation assay

For the DNA relaxation assay, supercoiled plasmid DNA was prepared using the Nucleobond kit (Bioké). 500 ng of supercoiled plasmid DNA was incubated with 0.12 units of Topoisomerase I (New England Biolabs) in the buffer supplied by the manufacturer with increasing amounts of SsbB. A total reaction volume of 25 µl was incubated at 37°C for 30 mins and stopped by the addition of 10 mM EDTA and incubation at 65°C for 20 mins. The samples were run on a 1% agarose gel at 100 V for 1 hr and then stained in buffer containing ethidium bromide for 30 mins and visualized using an UV gel documentation system (Bio-Rad).

## Results

### Sequence analysis of SsbB

**Figure 1:**
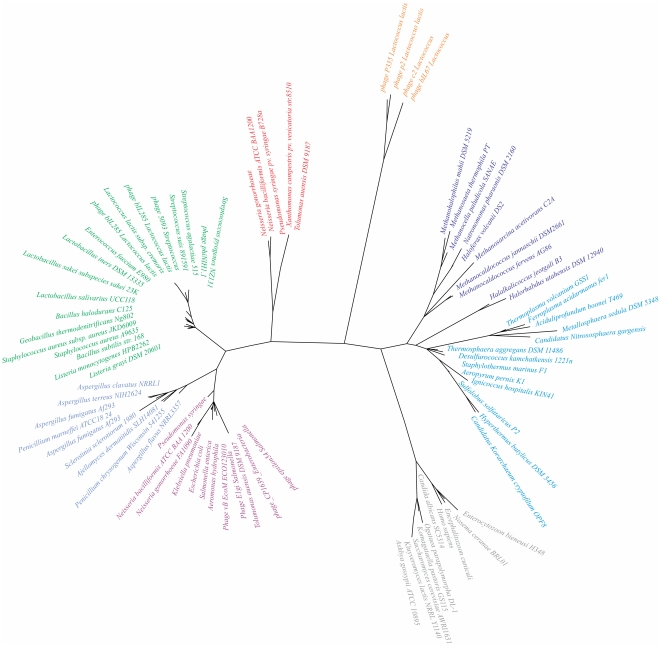
Unrooted phylogenetic tree of the ssDNA-binding proteins. The SSB phylogeny was reconstructed using sequences representative of the different SSB families [28] as described in Materials and Methods. SSB proteins related to *N. gonorrhoeae* SsbB found in Genetic Islands or Integrated Conjugative Elements are shown in red. Other colours indicate: Eukaryotes (grey), Crenarchaea (blue), Euryarchaea (dark purple), mitochondria (light blue), Gram-negative bacteria (light purple), Gram-positive bacteria (green), Lactococcus phages (orange).

The SSB encoded within the GGI was previously annotated as SsbB on the basis of 28% identity to *Xylella fastidiosa* XF1778 SsbB [26]. As most SSBs, SsbB contains an N-terminal conserved OB fold in the region between residues 5 and 108 [11]. SsbB contains a relatively short disordered acidic C-terminus and the OB fold of SsbB shares only relatively low sequence similarity with other SSBs (See Figure S1). Analysis of the genetic surroundings of the closest homologs of SsbB revealed that SsbB is located in a cluster of homologous genes that is found in several proteobacteria (See Figure S2). Remarkably, this cluster contains the DNA partitioning proteins ParA and ParB, four conserved hypothetical proteins containing different domains of unknown functions and a Topoisomerase. Three of the four conserved proteins contain conserved domains of unknown function (DUF2857, DUF1845 and DUF 3158). These clusters are often found at the borders of large genetic islands, like the PAGI-3(SG), PAGI-2(C) and the clc-like genetic islands found in *Pseudomonas aeruginosa* and other organisms [34], [35]. The cluster found within the GGI contains next to SsbB, the ParA and ParB proteins, the Topoisomerase, the proteins with the DUF 2857 (YfeB) and the DUF1845 (Yfb) domain. YfeA, Yfd and Yfa show only very little to no homology to the other two conserved hypothetical proteins. Other clusters in which some of the proteins are missing are also identified in other species. To determine the relation of these SSBs encoded within genetic islands with other SSBs, a phylogenic tree was constructed. The sequences used to generate this tree were based on 78 ssDNA-binding protein sequences, which were recently used to identify different subfamilies of eukaryotic, crenarchaeal, euryarchaeal, mitochondrial, gram-negative and gram-positive bacteria SSBs and a subfamily proposed to consist exclusively of the proteins from lactococcal bacteriophages [28]. The sequences of proteins with strong homology to *N. gonorrhea* SsbB, encoded within genetic islands and their chromosomal homologs were included in this set, and a phylogenetic tree was created (See Figure 1). Our phylogenetic analysis demonstrated that the *N. gonorrhoeae* SsbB-like proteins form a separate cluster of SSB proteins. The phylogenetic distance from other SSBs and the genetic surroundings suggests that these proteins have evolved within a cluster of proteins to form a novel class of SSBs with a possible role in partitioning, stability or transport of the DNA of the genetic islands in which they are encoded. To further characterize a member of this protein family, the operon structure and the expression levels of *ssbB* were determined.

### *ssbB* is encoded within an operon that is expressed in *N. gonorrhoeae*

**Figure 2:**
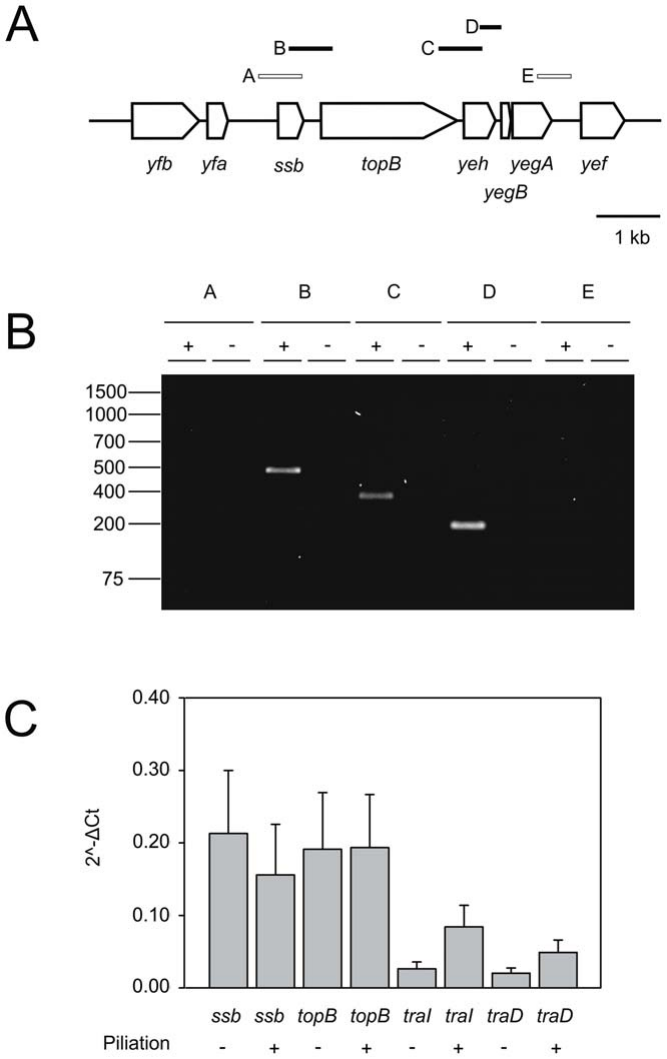
Analysis of the transcription of the *yfa-yef* region. Reverse transcriptase was used to map the operon structure of the *ssb-yegA* region within the GGI of *N. gonorrhoeae* strain MS11. A) Schematic representation of the *yfa-yef* region of the GGI. Genes are indicated by arrows and the expected PCR products by lines over the genes. Primer combinations for which a PCR product was obtained are indicated by black boxes and primer combinations for which no PCR product was obtained are indicated by white boxes. B) Operon mapping of the *ssb-yegA* operon. Transcripts were determined by PCR. (+) indicates reactions on cDNA created in the presence of reverse transcriptase and (−) indicates reactions on cDNA created in the absence of reverse transcriptase. C) Quantitative gene expression levels of *ssbB*, *topB*, *traI* and *traD* of piliated and non-piliated *N. gonorrhoeae* strains were determined by qRT-PCR. The graph shows the mRNA levels as comparative gene expression after normalizing each gene to *secY*. Values depict means ± standard deviation of six biological replicates.

Currently no information is available about the expression and function of the SsbB protein encoded within the GGI or any of its close homologs. The *ssbB* gene is located between several genes transcribed in the same direction (Figure 2A). Homologs of the ParA and ParB proteins, the topoisomerase, and the proteins with the DUF2857 (YfeB) and the DUF1845 (Yfb) domains are conserved within the SsbB homologs encoded within genetic islands. (See Table S5). The *yegA* gene is followed by a previously unnamed gene (annotated as NgonM_04872 in the MS11 whole genome shotgun sequence) which encodes a 149 amino acids long conserved hypothetical protein with a DUF3577 domain. This gene was named *yef*. In general, intergenic regions between the open reading frames (ORFs) of these genes are small (Table S5), suggesting transcription in polycistronic messengers. To analyze the transcriptional linkage of these genes, reverse transcription PCR (RT-PCR) was performed with primer pairs spanning different intergenic regions (Figure 1B). Successful amplification by these primer pairs was confirmed on chromosomal DNA (data not shown). No amplification products were detected in control reactions in the absence of reverse transcriptase (Figure 2B). The RT-PCR analysis demonstrated that the *ssbB*, *topB*, *yeh*, *yegB* and *yegA* genes form an operon (Figure 2A). It further showed that the *parA*, *parB*, *yfeB* and *yfb* genes, although they are often found genetically linked to *ssb*, are not encoded in the same operon. In a next step we set-out to determine whether any possible regulation of the operon could be identified. The first operon of the GGI containing the *traI* and *traD* genes which encode proteins involved in targeting the secreted DNA to the secretion apparatus is upregulated in piliated cells compared to non-piliated cells [36]. To determine the expression levels of the *ssbB* gene and to test whether a similar difference could be observed in the expression of the *ssbB-yegA* operon, a qualitative real time PCR (qRT-PCR) using primers designed against the *ssbB*, *topB*, *traI* and *traD* genes and against the *secY* gene as a control was performed on mRNA isolated from piliated and non-piliated strains (Figure 2C). The qRT-PCR revealed relatively low levels of transcription compared to the transcript containing the *secY* gene but higher levels of transcription than the *traI* and *traD* genes. However, no differences in the expression levels of the *ssbB* and *topB* genes were observed between piliated and non-piliated cells.

### *N. gonorrhoeae* SsbB does not complement the *E. coli ssb* mutant

Since we have determined that the *ssbB* gene is expressed in *N. gonorrhoeae*, we set-out to further characterize SsbB. Many SSBs, independently of whether they were encoded on plasmids or on the chromosome [31], [37], [38], [39] have been shown to be able to complement the essential chromosomal *E. coli ssb* gene for cellular viability. To test whether SsbB could complement the *E. coli* SSB, *ssbB* was cloned downstream of a *lac* promoter in an *E. coli* expression vector, and tested using a complementation assay described previously [31]. Remarkably, SsbB was not able to complement the *E. coli ssb* mutation. Since this strongly differs from other characterized SSBs, and none of the members of this cluster of SSB proteins have been studied, we further set out to characterize the function of *N.gonorrhoea* SsbB.

### Overexpression, purification and determination of the oligomeric state of SsbB

SsbB was expressed in *E. coli* as the native protein and with N-terminal OneSTrEP- and His-tags. The three proteins were purified to homogeneity (>99% purity as assayed by Silver staining, data not shown) with yields of 1.7, 3.5 and 10 mg/g wet cells for native, His-tagged and OneSTrep-tagged SsbB respectively. Analysis by gel filtration chromatography revealed single peaks for the WT and N- His- and OneSTrEP tagged proteins respectively, indicating that all three proteins form stable tetramers (data not shown). Attempts to destabilize the tetramer by incubations at increasingly higher temperatures or with increasing concentrations of chaotrophic agents like guanidinium and urea led to aggregation of the protein before any monomeric proteins could be detected (data not shown), demonstrating that SsbB forms a stable tetramer that is difficult to dissociate.

### SsbB binds binds to fluorescently and radioactively labeled ssDNA with high but different affinities

**Figure 3:**
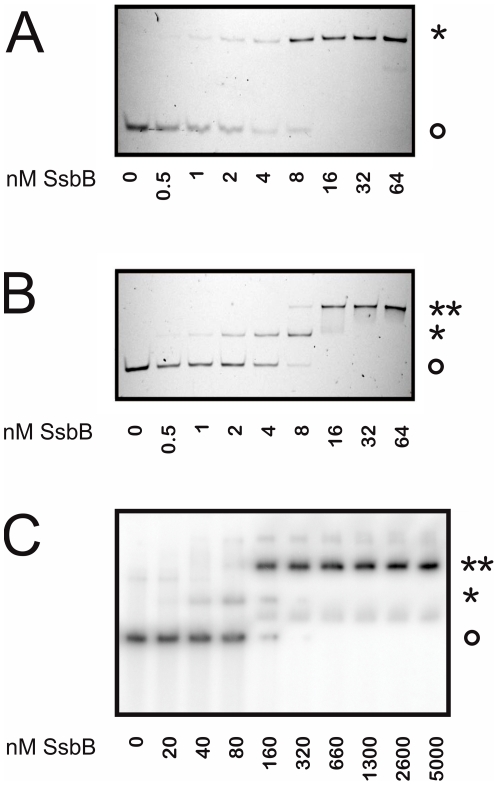
Analysis of the binding mode of SsbB by electrophoretic mobility shift assays. A) 8 nM of fluorescently labeled dT_35_ and B) dT_75_ oligonucleotides were incubated in SBA buffer (10 mM NaOH, 2 mM EDTA, pH 7.5) with increasing concentrations (0–64 nM) tetrameric SsbB. The reactions were separated by polyacrylamide gel electrophoresis and were visualized using a LAS-4000 imager (Fujifilm). C) 2 nM of a ^32^P- labeled dT_75_ oligonucleotide was incubated with increasing concentrations (0–5000 nM) tetrameric SsbB in SBA buffer. The reactions were separated by polyacrylamide gel electrophoresis and were visualized by autoradiography. The circle (o) indicates the free oligonucleotide, while one (*) or two (**) asterixes represent oligonucleotides bound with one or two SsbB tetramers.

To determine whether SsbB binds ssDNA, fluorescently Cy3-labeled dT_35_ and dT_75_ oligonucleotides were incubated with increasing amounts of purified SsbB (Figure 3A and 3B) and used in electrophoretic mobility shift assays (EMSA). The 35-mer Cy3-labeled oligonucleotide showed a single mobility shift upon binding to SsbB (Figure 3A). Binding occurred with a stoichiometry of one dT_35_ oligonucleotide per tetramer. A similar experiment performed with the Cy3-labeled 75-mer showed two complexes with different mobilities (Figure 3B). The first complex was formed at a (SsbB)_4_/dT_75_ ratio of 1 and the second complex was formed at an (SsbB)_4_/dT_75_ ratio of 2, demonstrating that the first complex contains one SsbB tetramer and the second complex contains two tetramers bound to the dT_75_ nucleotide. Stoichiometric binding was observed at 8 nM for both the dT_35_ and dT_75_ oligonucleotides, which is a higher affinity then is generally reported for other SSBs in EMSAs [40], [41]. We therefore performed a similar experiment with a radioactively labeled dT_75_ oligonucleotide (Figure 3C). Again two different complexes, representing one and two SsbB tetramers bound to the T_75_ oligomer were observed. The complexes were however formed at a concentration of approximately 100 nM (SsbB)_4_, which is similar to affinities observed for other SSBs, but 10-fold lower than the affinity observed for the fluorescently labeled oligonucleotide. This demonstrates that the presence of a fluorescent label on an oligonucleotide can strongly influence the binding properties. Since the observed affinity was affected by the presence of the fluorescent probe, we also studied the effects of other components on the observed affinity. No large differences were observed when the binding reactions were performed using either SBA, Tris-HCl or Tris-Acetate based buffers supplemented with either 0 or 10 mM MgCl_2_ and/or 10, 200 or 500 mM NaCl. To compare the effects of N-terminal tags on ssDNA binding, the EMSAs described above were also performed with native SsbB, His-tagged SsbB and OneSTrEP-tagged SsbB. No differences were found between native SsbB and OneSTrEP-tagged SsbB, but His-tagged SsbB bound ssDNA with a lower affinity (data not shown). Therefore, the following experiments were only performed using native or OneSTrEP-tagged SsbB.

### Fluorescence characterization of the binding of SsbB to ssDNA

**Figure 4:**
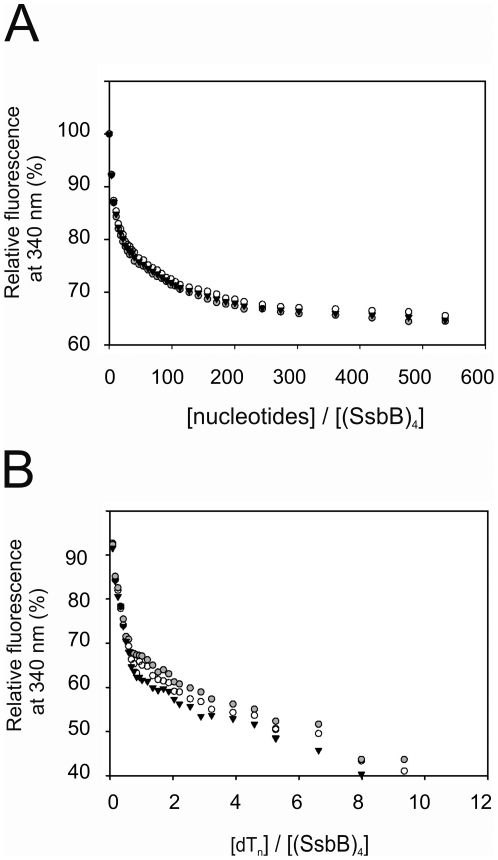
Analysis of the minimal binding frame of SsbB by electrophoretic mobility shift assays. A) Determination of the minimal binding frame of one SsbB tetramer. Each binding reaction contained 1 µM (SsbB)_4_ and 5 µM dT_n_ of different lengths (6–35 nucleotides) and was performed in SBA buffer. The reactions were analyzed by polyacrylamide gel electrophoresis and SsbB was later visualized by Coomassie staining. B) Determination of the minimal binding frame of two SsbB tetramers. The binding reaction contained 1 µM (SsbB)_4_ and 0.25 µM dT_n_ of different lengths (67–74 nucleotides) and was performed in SBA buffer. The reactions were analyzed by polyacrylamide gel electrophoresis and SsbB was later visualized by Coomassie staining. The square (□) indicates the free SsbB tetramer, while one (*) or two (**) asterixes represent an oligonucleotide bound to one or two SsbB tetramers.

To further study the binding behavior of SsbB to ssDNA, fluorescence titrations were performed. In these experiments, the quenching of the two tryptophans of SsbB upon binding was used to analyze ssDNA binding. Fluorescence titrations with poly(dT) under low (20 mM NaCl), medium (100 mM NaCl) and high (500 mM NaCl) salt conditions and in the presence of 10 mM MgCl_2_ are shown in Figure 4A. The average length of the poly(dT) was approximately 1000 bases as estimated by agarose gel electrophoresis. When binding to poly(dT), the intrinsic tryptophan fluorescence of SsbB decreases with only 35%. Remarkably, the fluorescence quenching is lower than normally seen for other SSBs and the quenching is not dependent on either the salt (see Figure 4A) or the Mg^2+^ concentration (data not shown). Unfortunately, no suitable fit was obtained when the binding curves were fitted to the model of Schwarz and Watanabe [42] (Personal communication, Peter Lens, Philipps-Universität Marburg). In a subsequent experiment, titrations were performed with dT_n_ oligomers of fixed lengths. Titrations nucleotides with lengths (n) of 25, 35 and 45 are shown in Figure 4B. These data show a biphasic curve. The initial phase shows that SsbB binds with high affinity to these oligonucleotides with a stoichiometry of 1 oligonucleotide per SsbB tetramer. The initial phase results in approximately 35% quenching, similar to what was observed for the poly(dT). The second phase represents a second binding event with much lower affinity. These data thus might suggest that SsbB, similarly to what has been observed for *E.coli* SSB [12], binds initially one oligonucleotide per SsbB tetramer, most likely in a (SSB)_35_ like manner, followed by a second binding event that occurs with a much lower affinity.

### Determination of the minimal binding frame for one or two SsbB tetramers

**Figure 5:**
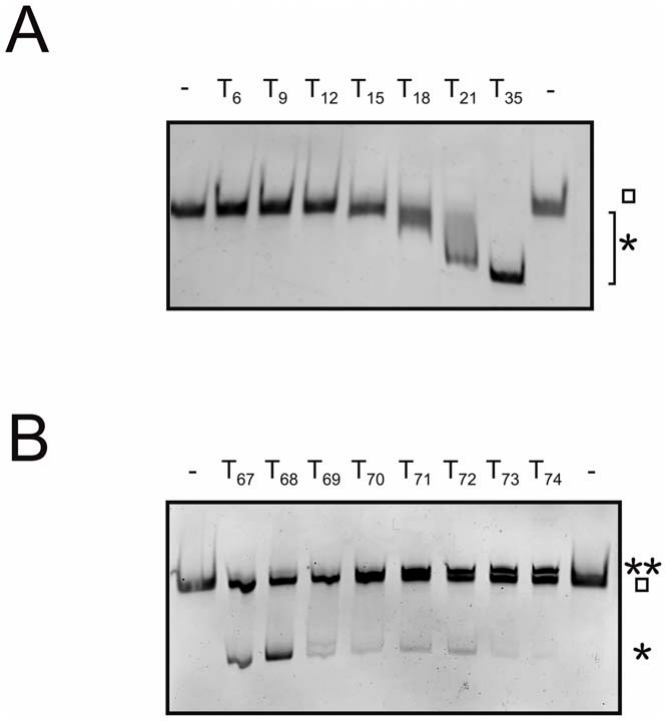
Fluorescence titrations of SsbB. A) 0.4 µM SsbB was titrated with increasing concentrations of poly(dT) in a buffer containing 20 mM Tris pH 7.5 and either 20 mM NaCl (open circles), 200 mM NaCl (close circles) and 500 mM NaCl (closed triangles). B) 0.4 µM SsbB was titrated with increasing concentrations of dT_n_ of 25 (closed circles), 35 (open circles) and 45 (closed triangles) nucleotides in a buffer containing 20 mM Tris pH 7.5 and 200 mM NaCl.

Since we were unable to determine the binding frame using fluorescence titrations, EMSAs were performed with poly(dT) oligonucleotides with different lengths (Figure 5A). In these experiments, the gels were coomassie stained to detect the SsbB protein. These experiments were performed in an excess of oligonucleotides and showed a small mobility shift for 15 nucleotides and larger shifts for oligonucleotides of increasing lengths. This demonstrated that SsbB can bind 15 nucleotides and longer. To determine the minimal length required to bind two SsbBs, EMSAs were performed with even longer oligonucleotides (Figure 5B). When these EMSAs were performed at low protein to nucleotide ratios ((SsbB)_4_/dT_n_<1) only binding of one tetramer per dT_n_ was observed. Further experiments were performed at a tetrameric SSB to nucleotide ratio of 4 ((SsbB)_4_/dT_n_ = 4). Upon increasing the length of the added oligonucleotide, lengths smaller than 70 nucleotides resulted in a shift to a faster mobility as compared to the free protein indicating binding of one SsbB tetramer. In contrast, at oligonucleotide lengths larger than 70 nucleotides a small shift was observed to a slower mobility, indicating the binding of two SsbB tetramers. These experiments demonstrated that the minimal binding frame for two SsbB tetramers is 70 nucleotides.

### SsbB binding to ssDNA visualized by atomic force microscopy

**Figure 6:**
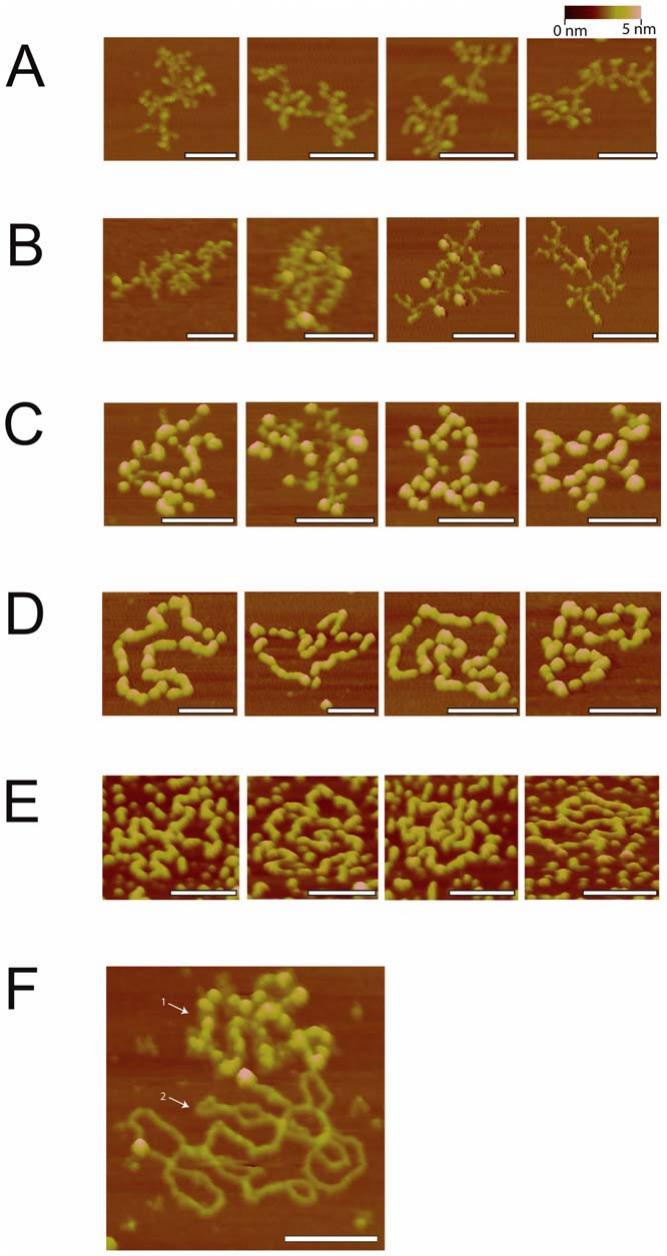
SsbB binding to M13 ssDNA visualized by Atomic force microscopy. A selection of AFM images, zoomed to display one DNA molecule or complex per image. The scale bar in all pictures equals 100 nm. These images were made for SsbB-ssDNA complexes at concentration ratios (R, corresponding to tetramer/nucleotides) of (A) 0, (B) 1/707, (C) 1/354, (D) 1/88 and (E) 1/44. (F) SsbB binds only to ssDNA (indicated by 1) and not to dsDNA (indicated by 2). The two bound proteins on the dsDNA are probably not SsbB, as indicated by their larger apparent volume, but impurities present in the M13 preparation.

AFM experiments were performed in air to analyze the architecture of SsbB-ssDNA complexes at a single molecule-level (Figure 6). SsbB protein was incubated with M13 ssDNA, which is a 6407 nt-long circular DNA molecule. Images were recorded of deposited reactions with concentration ratios (R) ranging from 1/707 to 1/44 (corresponding to tetramer/nucleotides). In order to improve the adsorption of the ssDNA molecules and complexes, the trivalent cationic polyamine spermidine was included in the reaction mixtures, as described before [21]. Adsorbed unbound ssDNA molecules visualized with AFM, appear condensed because of hairpins and other secondary structures that are formed between complementary regions (Figure 6A). At low ratios, SsbB tetramers bind the DNA apparently randomly, observed as individual “blobs” on the nucleoprotein complexes (Figure 6B and C). Tetramers do not bind in arrays or clusters, but are rather distributed independently over the ssDNA molecules. This might suggest that under these conditions SsbB binds preferentially to DNA regions without secondary structure, and is initially excluded from condensed regions. At higher R, DNA molecules are saturated by SsbB protein, thereby resolving the condensed ssDNA structures (Figure 6D and E). As observed for *E.coli* SSB, the co-existence of different types of structures (quasi-naked ssDNA, more or less saturated complexes) was observed in the same deposition. This indicates that there might be some limited cooperativity in the SsbB-ssDNA interaction, upon binding to longer ssDNA molecules [21]. Evidence is also provided that SsbB binds specifically to ssDNA, and not to dsDNA. A deposition of a reaction mixture containing both types of DNA visualized a saturated SsbB-ssDNA complex adsorbed next to an unbound dsDNA molecule (Fig. 6F).

### SsbB has no effect on DNA secretion or uptake

**Figure 7:**
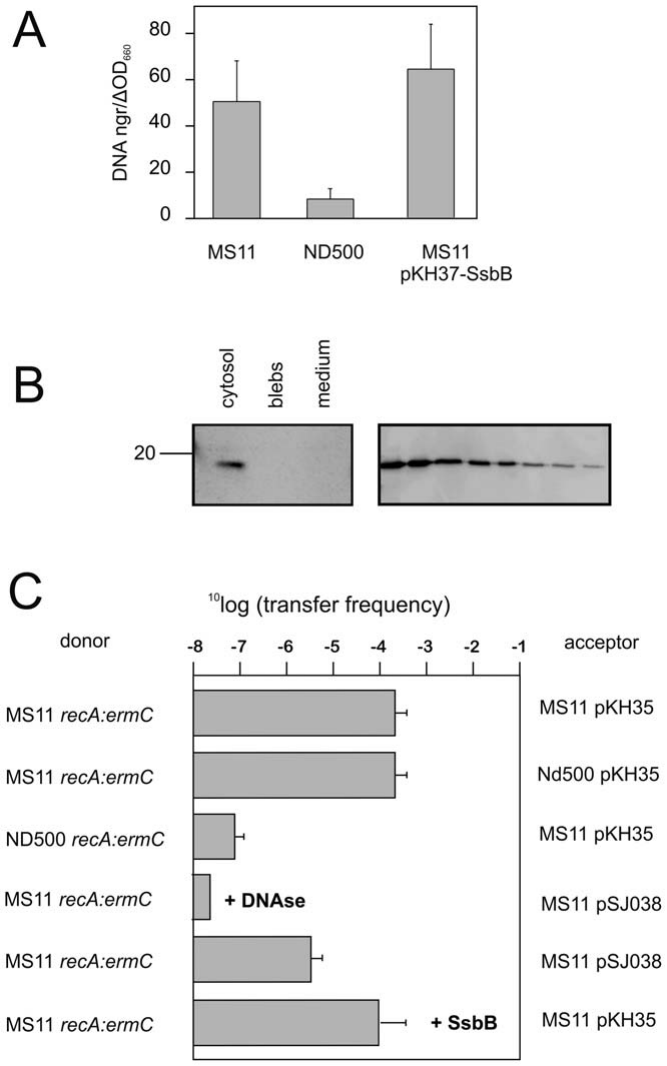
*In vivo* functional analysis of SsbB in *Neisseria gonorrhoeae*. A) DNA secretion assay with fluorimetric detection of the secreted DNA in the culture supernatant. MS11 is the wild type strain which contains the GGI and ND500 is the MS11 strain in which the GGI was deleted. This strain does not secrete DNA into the medium. Strain MS11 transformed with plasmid pKH37-SsbB expresses SsbB from an inducible *lac* promoter. Results depicted are the average of at least 3 independent experiments. (B) Western blot using anti-Strep II antibody to detect the secretion of SsbB in the medium. Different fractions of the *Neisseria gonorrhoeae* strain SJ023-MS overexpressing N-terminal OneSTrEP-tagged SsbB from an inducible *lac* promoter were isolated and run on a 15% SDS-PAGE gel. The different lanes are representative of the cytosolic, blebs and the medium fractions, isolated from 240 µl, 20 ml and 2 ml of a logarithmically growing culture of OD_600_∼0.5. (C) Co-culture DNA transfer assay to determine the effect of SsbB on the DNA uptake efficiency. Donor and recipient strains were mixed and grown together at 37°C for 5 hrs and plated on selective media. The donor strains contain the erythromycin marker in the *recA* gene and the recipients contain the pKH37 or pSJ038 plasmids that contain the chloramphenicol marker and are integrated into the chromosome between the *aspC* and *lctP* genes. Vector pSJ038 is derived from pKH37 and expresses SsbB from an inducible *lac* promoter. The transfer of the erythromycin was measured as transfer frequency (CFU of transconjugants per CFU of donor). The values are the average from three independent experiments. It is indicated when purified SsbB (3.5 µM) and DNase I were added to the medium.

Since it was demonstrated that SsbB is expressed and forms an active ssDNA binding protein, we commenced to study possible functions of SsbB. SsbB is encoded within the GGI that encodes a T4SS involved in the secretion of ssDNA into the medium. The ssDNA binding protein VirE2 encoded by the *A. tumefaciens* T4SS is transported to the recipient cells [8] where it helps in importing the bound single stranded DNA [10]. It also has previously been proposed that the SSB encoded within clc-like elements might be involved in DNA transport [43]. DNA secretion studies demonstrated that deletion of *ssbB* had no effect on the secretion of ssDNA (Pachulec, manuscript in preparation). To test whether overexpression of SsbB had any effect on ssDNA secretion, WT or OneSTrEP-tagged SsbB expressed from an inducible *lac* promoter was inserted into the chromosome of *N. gonorrhoeae* strain MS11. DNA secretion assays showed that there was no significant effect of SsbB overexpression on DNA release (Figure 7A). To test whether SsbB might be secreted, different fractions were isolated, and compared to an isolated cytosolic fraction. The medium was concentrated by trichloroacetic acid (TCA) and the outer membrane derived vesicles, called blebs [44], were concentrated by ultracentrifugation respectively. OneSTrEP-tagged SsbB could only be detected in the cytoplasmic fraction (Figure 7B). Western blotting with purified OneSTrEP-tagged SsbB showed that the detection limit is 50 fmol (corresponding to 1 ng or 10 µl of 5 nM solution, data not shown). In a further attempt to detect SsbB, OneSTrEP-tagged SsbB was purified from cells and medium using a Strep-tactin Sepharose column, but again significant amounts of SsbB could be purified only from the cytosolic fraction, but not of the medium fraction (data not shown). It is concluded that One-Strep-tagged SsbB is not secreted via the T4SS at significant levels.

Several SSBs like YwpH of *Bacillus subtilis* [2] and SsbB of *Streptococcus pneumoniae* [3] play an important role in DNA uptake and competence. To test whether SsbB might play a similar role, the effect of SsbB on the efficiency of DNA uptake by *N. gonorrhoeae* was tested in co-culture experiments (See Figure 7C). In these experiments, strains in which the *recA* gene is disrupted by an erythromycin marker to ensure unidirectional transfer of DNA were used as donor strains, whereas strains with a chlorampenicol marker were used as acceptor strains. Similar to previous observations, transfer of chromosomal markers increased strongly in strains containing the GGI, whereas the transfer decreased in strains not containing the GGI [25]. Similar transfer rates were observed when the transfer frequencies of chromosomal markers to either acceptor strains with or without the GGI were determined. Transfer of the markers was abolished when DNase I was added to the medium, but the addition of high concentrations of SsbB (3.5 µM) to the medium had no effect. When SsbB was overexpressed in the acceptor strain, a lower transformation rate was observed. Therefore overexpression of SsbB either affects DNA uptake, DNA stability in the acceptor strain, or the efficiency of recombination. It has previously been shown that SSB overexpression could have a negative effect on RecA recombinase activity [45]. Thus these data show that SsbB has no influence on ssDNA secretion and/or DNA uptake.

### SsbB stimulates topoisomerase activity

**Figure 8:**
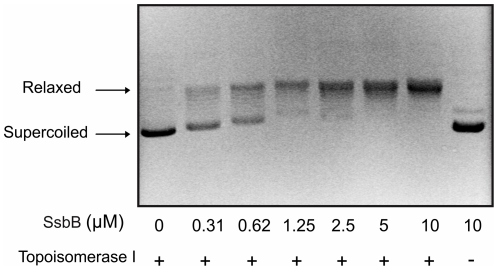
SsbB stimulates *E. coli* Topoisomerase I activity. Supercoiled plasmid DNA was incubated with 0.12 units of topoisomerase and with increasing amounts of purified SsbB, as indicated. Reactions were carried out at 37°C for 30 min. DNA was resolved on a 1% agarose gel and stained with ethidium bromide. Arrow heads indicate the relaxed and supercoiled forms of plasmid.

Since SsbB does not affect DNA secretion or uptake, further possible functions of SsbB were studied. In the GGI, *ssbB* is co-transcribed with the topoisomerase I, *topB*. It has been previously shown that other SSBs, like the SSBs of *E. coli* and of *Mycobacterium tuberculosis* could stimulate *E. coli* topoisomerase I activity [46]. It was shown that these stimulating effects occurred by enhancing DNA binding to toposiomerase I, and not via any direct interaction between the SSB and the topoisomerase I. SsbB strongly stimulated the activity of *E. coli* topoisomerase in a concentration dependent manner (Figure 8). This demonstrates that SsbB can stimulate a heterologous DNA processing enzyme.

## Discussion

Within this study we have found that the GGI encodes a conserved cluster of genes also found in other genetic islands. This cluster is often found in integrated conjugative elements like the PAGI-3(SG), PAGI-2(C) and the *clc*-like genetic islands found in *Pseudomonas aeruginosa* and other organisms, and consists of a core set of 8 genes, which are generally transcribed in the same direction, and consists of the partitioning proteins ParA and ParB, four conserved hypothetical proteins containing respectively a DUF2857, no DUF, a DUF1845 and a DUF 3158 domain, a ssDNA binding protein and a topoisomerase I. Similar to what is observed for the GGI, these clusters are often located near the border of the integrated element. Two of the eight proteins, the protein that did not contain a conserved DUF and the protein with a DUF3158 domain could not be identified within the GGI. The integrated conjugative elements in which this cluster is found often contain a type IV secretion system involved in conjugation of the integrated element. This occurs after excision of the element from the chromosome, and the generation of a circular intermediate. Indeed it has been proposed that such a cluster found in the *clc* genetic island of *Pseudomonas* sp. strain B13 might play a role in preparing the DNA for conjugal transfer [43], possibly by stabilization of the circular intermediate or targeting the DNA to the type IV secretion system. The GGI is maintained within the chromosome of *N. gonorrhoeae*. It is flanked by a perfect and an imperfect *dif* site. When repaired, the presence of two correct *dif* sites causes excision of GGI from the chromosome by the XerCD recombinase [47]. The excised circular GGI can only be detected transiently, but even the transiently present circular GGI might transfer from one cell to another. Mutagenesis of ParA within the GGI abolished the secretion of ssDNA [26], further suggesting a role for the ParA-TopB genetic cluster in the maintenance or transport of ssDNA.

To further functionally characterize SsbB, it was purified to homogeneity. Similar to many other SSBs, SsbB was shown to form a stable tetramer. The tetramer bound ssDNA with high affinity, characterized by an equilibrium dissociation constant of 100 nM. Remarkably, SsbB bound with at least 10-fold higher affinity to Cy3-labeled oligonucleotides, demonstrating that fluorescent labels can strongly influence the binding affinity. Fluorescently labeled oligonucleotides are currently widely used, and our data shows special care should be taken when they are used to directly determine binding affinities.

A combination of EMSAs, fluorescence titrations and atomic force microscopy were used to characterize the binding of SsbB to ssDNA. This demonstrated that the oligonucleotide length required for SsbB binding was approximately 15 nucleotides, which is similar to the binding frames of the SSBs of *E. coli* and *Mycobacterium tuberculosis* that vary between 15 and 17 nucleotides [48], [49]. Fluorescence titrations demonstrated that SsbB binds first to one oligonucleotide after which a second oligonucleotide can only bind to the same SsbB with lower affinity. A similar negative cooperativity was observed for *E.coli* SSB [12],[50]. Titrations to determine the oligonucleotide length to which two SsbBs could bind showed that a second SsbB tetramer could only bind if the ssDNA was longer than 70 nucleotides. Indeed, many different SSBs can bind with 2 SSB tetramers to an oligonucleotide of 75 nucleotides at low salt or low Mg^2+^ concentrations [12]. Generally, these SSBs, like for example the *E. coli* SSB, bind DNA with two of the OB folds occluding approximately 35 nucleotides in a highly cooperative mode. At higher salt or Mg^2+^ concentrations, the binding mode changes to a mode with lower co-operativity where the ssDNA is bound to four OB folds occluding approximately 65 nucleotides. In this mode only one SSB tetramer can bind to an oligonucleotide of 75 nucleotides [12]. Remarkably, within our experiments we have not observed that SsbB binding to ssDNA was sensitive to either salt or Mg^2+^ concentrations. Using atomic force microscopy, we have also studied binding to longer DNA fragments. SsbB tetramers bound distributed independently over the ssDNA molecules. This might suggest that SsbB binding is initially excluded from condensed regions and that SsbB initially only binds to DNA regions without secondary structure. Regions with higher secondary structure are only resolved at higher SsbB concentrations. SsbB was expressed only at low levels under normal growth conditions, suggesting that SsbB under these conditions either binds distributed evenly over exposed ssDNA stretches, or is specifically targeted to certain regions by other proteins.

Although SsbB functions in many respects similar to other SSBs, SsbB could not complement the *E.coli ssb* mutant, even when overexpressed to high levels. We therefore set out to find a specific function for SsbB. First, the role of SsbB in ssDNA secretion was studied. VirE2, a ssDNA binding protein encoded on the *A. tumefaciens* Ti plasmid is necessary for transport of the T-DNA to the plant cell nucleus. VirE2 is transported directly to the target cell, where it binds and protects the ssDNA [51]. It was demonstrated that the binding of the transported VirE2 to the ssDNA pulls the DNA into the target cell [9]. Before transport to the target cell, VirE2 is kept transport competent by VirE1 [8]. No homolog of VirE1 could however be detected within the GGI, and neither deletion of the *ssbB* gene nor the overexpression of SsbB affected ssDNA secretion. SsbB could not be detected in the medium isolated from strains involved in ssDNA secretion via the type IV secretion system. Also the addition of purified SsbB to the culture supernatant at concentrations 1000 fold higher then detected in the medium did not affect the GGI dependent transfer of chromosomal markers. This makes it unlikely that SsbB either function inside the cell to assist the transport of DNA, or is secreted into the medium where it could assist the transport of the ssDNA. Another possibility studied is that SsbB functions not in the process of export of ssDNA, but in the process of the uptake of ssDNA. If SsbB is involved in competence, it is expected that the presence of SsbB increases the transformation efficiency. Surprisingly, when SSB was overexpressed in the recipient cell, the transformation efficiency was reduced. Most likely the overexpression of SsbB interferes with the activity of RecA in the recombination process [45], [52]. It is therefore concluded that SsbB is not involved in DNA secretion and uptake.

In general, these SSBs found in genetic islands might together with the topoisomerase I homologs serve to stabilize the circular form of the GGI when it has excised from the chromosome before it is re-integrated or exported. We demonstrate that SsbB, together with TopB forms an operon separately from the other genes found in the conserved cluster. This suggests that, although the partitioning protein ParA and the SSB are found in the same cluster they might function in different processes. The possible interaction with other proteins encoded within the GGI should be studied to further characterize the role of the SSBs within the genetic islands.

## Supporting Information

Figure S1 **Sequence comparison of different ssDNA binding proteins.** (*Escherichia coli* SSB (GenBank: AAA24649.1), the chromosomal SSB of *Neisseria gonorrhoeae* MS11 (GenBank: ZP_06132898.1), *Pseudomonas aeruginosa* SSB (GenBank: AAG07620.1), *Xylella fastidiosa* 9a5c SSB (NP_299066.1), *Bacillus subtilis* SsbA (ADM40106.1), *Streptococcus pneumoniae* SsbA (GenBank: ABJ55175.1), F-plasmid SSB (GenBank:NP_061439.1), *B. subtilis* SsbB (ADM39609.1), *S. pneumoniae* SsbB (GenBank: ABJ54110.1) and SsbB of *N. gonorrhoeae* MS11. Identical residues are highlighted in black, similar residues are highlighted in grey.
(DOCX)

Figure S2 **Comparison of the genetic environment of homologs of SsbB of** ***N. gonorrhoeae*** **reveals that the** ***ssbB*** **gene is located within a cluster conserved in several proteobacteria.** Shared synteny was determined and the figure was composed using the Absynte website (http://archaea.u-psud.fr/absynte). Homologous proteins are indicated using similar colors.
(DOCX)

Table S1 Organisms and corresponding accession numbers used to create the phylogenetic tree.
(DOCX)

Table S2 Strains used in this study.
(DOCX)

Table S3 Plasmids used in this study.
(DOCX)

Table S4 Primers used in this study.
(DOCX)

Table S5 Organization of the genetic cluster surrounding *N. gonorrhoeae* SsbB.
(DOCX)
