# Supplementary figures and images for: Characterization of the Single Stranded DNA Binding Protein SsbB Encoded in the Gonoccocal Genetic Island
Source: PLoS One. 2012 Apr 19;7(4):e35285. doi: 10.1371/journal.pone.0035285 (PMC3334931; doi:10.1371/journal.pone.0035285)

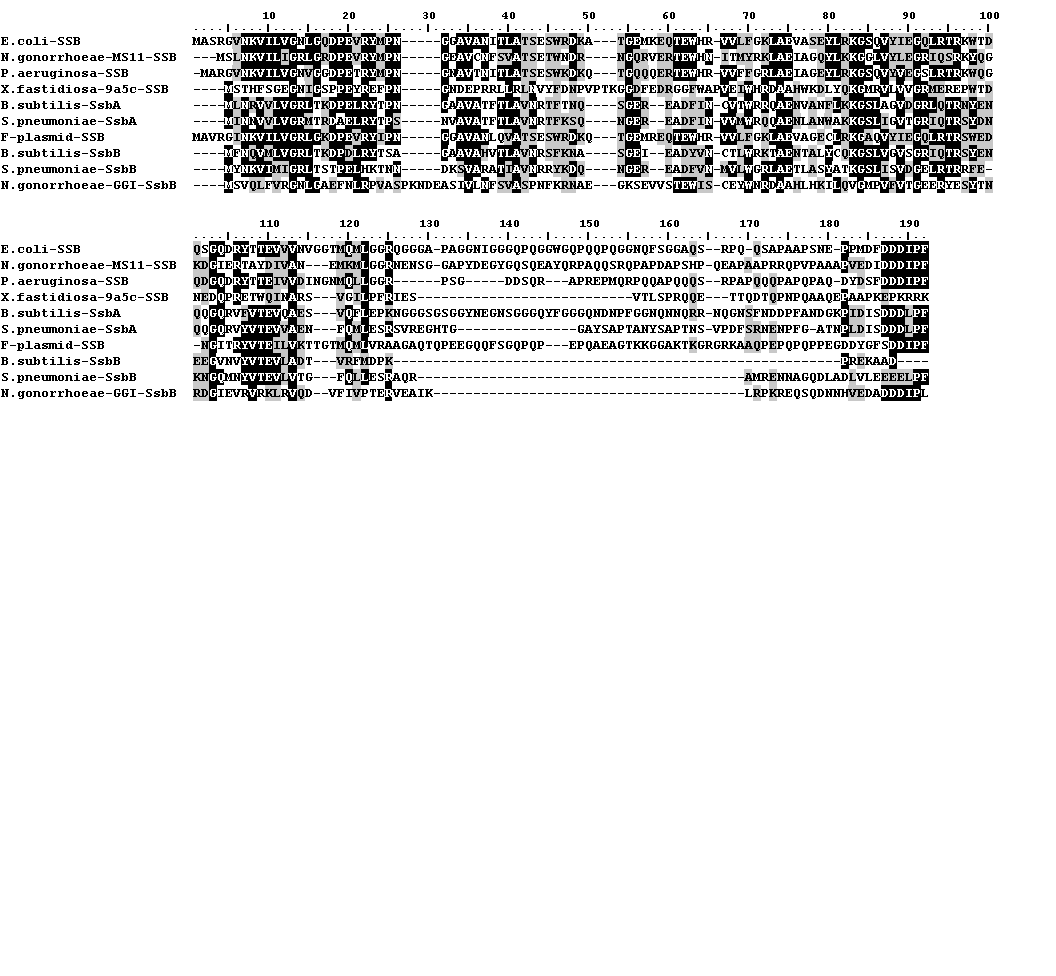

Supplement: Figure S1 — Sequence comparison of different ssDNA binding proteins. (Escherichia coli SSB (GenBank: AAA24649.1), the chromosomal SSB of Neisseria gonorrhoeae MS11 (GenBank: ZP_06132898.1), Pseudomonas aeruginosa SSB (GenBank: AAG07620.1), Xylella fastidiosa 9a5c SSB (NP_299066.1), Bacillus subtilis SsbA (ADM40106.1), Streptococcus pneumoniae SsbA (GenBank: ABJ55175.1), F-plasmid SSB (GenBank:NP_061439.1), B. subtilis SsbB (ADM39609.1), S. pneumoniae SsbB (GenBank: ABJ54110.1) and SsbB of N. gonorrhoeae MS11. Identical residues are highlighted in black, similar residues are highlighted in grey. (DOCX) [file pone.0035285.s001.docx]

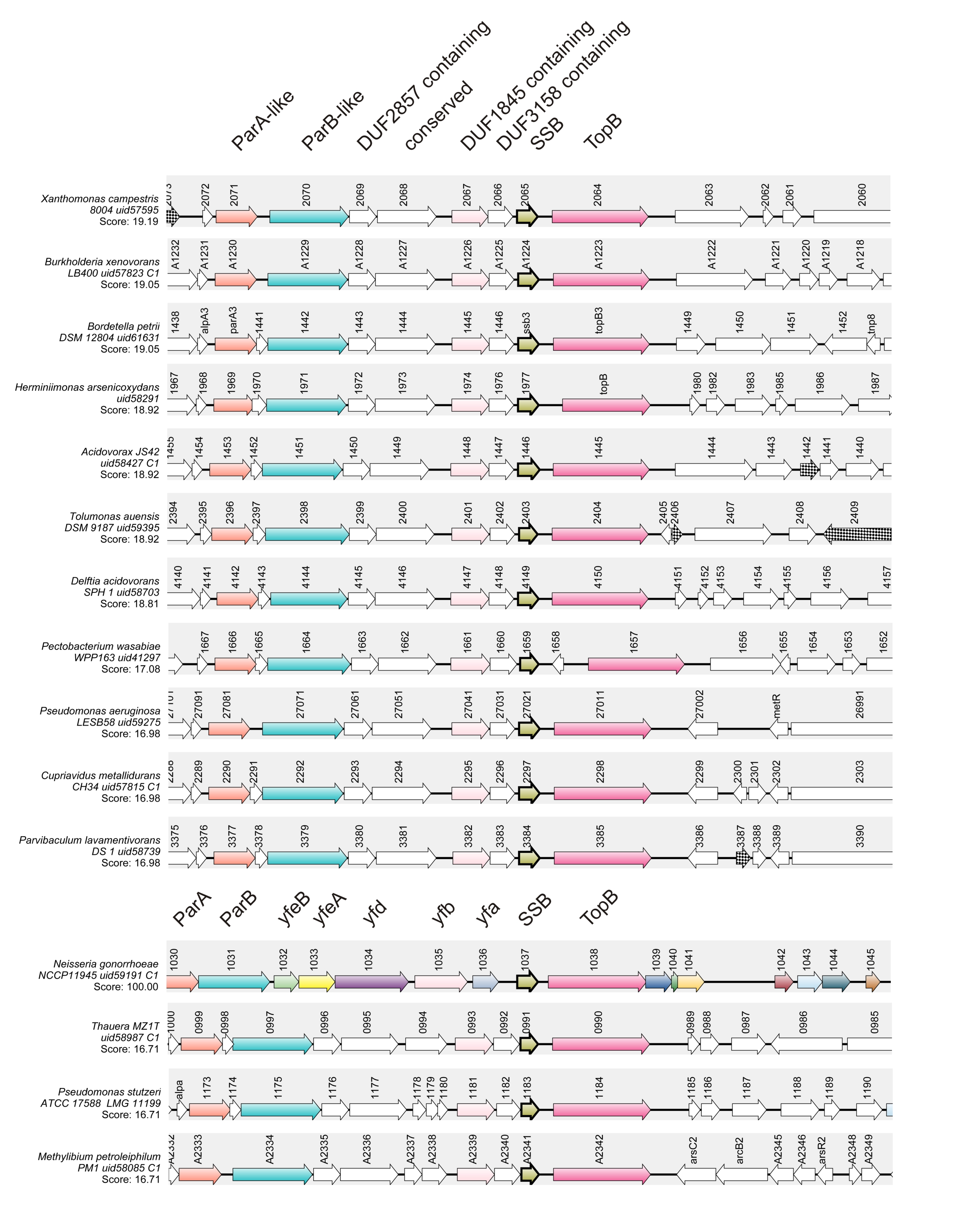

Supplement: Figure S2 — Comparison of the genetic environment of homologs of SsbB of N. gonorrhoeae reveals that the ssbB gene is located within a cluster conserved in several proteobacteria. Shared synteny was determined and the figure was composed using the Absynte website (http://archaea.u-psud.fr/absynte). Homologous proteins are indicated using similar colors. (DOCX) [file pone.0035285.s002.docx]
