# Supplementary material for: Characterization of the Single Stranded DNA Binding Protein SsbB Encoded in the Gonoccocal Genetic Island
Source: PLoS One. 2012 Apr 19;7(4):e35285. doi: 10.1371/journal.pone.0035285 (PMC3334931; doi:10.1371/journal.pone.0035285)
Supplement: Table S1 — Organisms and corresponding accession numbers used to create the phylogenetic tree. (DOCX) [file pone.0035285.s003.docx]

| **Organism** | **Accession number** |
| --- | --- |
| *Neisseria gonorrhoeae* | YP_002001662.1 |
| *Neisseria bacilliformis ATCC BAA1200* | ZP_08246626.1 |
| *Pseudomonas syringae pv. syringae B728a* | YP_234610.1 |
| *Xanthomonas campestris pv. vesicatoria str.8510* | YP364019.1 |
| *Tolumonas auensis DSM 9187* | YP0_02893586.1 |
| *Halalkalicoccus jeotgali B3* | ADJ15268.1 |
| *Halorhabdus utahensis DSM 12940* | YP_003129464.1 |
| *Natronomonas pharaonis DSM 2160* | YP_325819.1 |
| *Haloferax volcanii DS2* | YP_003534588.1 |
| *Methanosarcina acetivorans C2A* | NP_615554.1 |
| *Methanohalophilus mahii DSM 5219* | YP_003541205.1 |
| *Methanosaeta thermophila PT* | YP_843314.1 |
| *Methanocella paludicola SANAE* | YP_003358045.1 |
| *Methanocaldococcus jannaschii DSM2661* | NP_248153.1 |
| *Methanocaldococcus fervens AG86* | YP_003128067.1 |
| *Enterocytozoon bieneusi H348* | XP_002650118.1 |
| *Nosema ceranae BRL01* | XP_002996585.1 |
| *Encephalitozoon cuniculi* | GBM1_CAD25779.1 |
| *Homo sapiens* | NP_002936.1 |
| *Ogataea parapolymorpha DL-1* | EFW95224.1 |
| *Komagataella pastoris GS115* | XP_002489756.1 |
| *Candida albicans SC5314* | CaO19.9640 |
| *Saccharomyces cerevisiae AWRI1631* | NP_009404.1 |
| *Kluyveromyces lactis NRRL Y1140* | XP_451388.1 |
| *Ashbya gossypii ATCC 10895* | NP_985540.1 |
| *Aeropyrum pernix K1* | NP_147870.2 |
| *Ignicoccus hospitalis KIN41* | YP_001435540.1 |
| *Thermosphaera aggregans DSM 11486* | YP_003649650.1 |
| *Desulfurococcus kamchatkensis 1221n* | YP_002428356.1 |
| *Staphylothermus marinus F1* | YP_001041417.1 |
| *Hyperthermus butylicus DSM 5456* | YP_001013024.1 |
| *Candidatus Korarchaeum cryptofilum OPF8* | YP_001736534.1 |
| *Sulfolobus solfataricus P2* | NP_343725.1 |
| *Metallosphaera sedula DSM 5348* | YP_001190557.1 |
| *Aciduliprofundum boonei T469* | ZP_04873662.1 |
| *Thermoplasma volcanium GSS1* | NP_111755.1 |
| *Ferroplasma acidarmanus fer1* | ZP_05570389.1 |
| *Candidatus Nitrososphaera gargensis* | ADK25968.1 |
| *phage P335 Lactococcus lactis* | YP_809066.1 |
| *phage c2 Lactococcus* | NP_043533.1 |
| *phage bIL67 Lactococcus* | AAA74351.1 |
| *phage p2 Lactococcus lactis* | AAR14301.2 |
| *Penicillium marneffei ATCC18 24* | XP_002145522.1 |
| *Aspergillus terreus NIH2624* | EAU31510.1 |
| *Sclerotinia sclerotiorum 1980* | XP_001591996.1 |
| *Pyrenophora triticirepentis Pt1CBFP* | EDU40347.1 |
| *Aspergillus fumigatus Af293* | EAL91809.2 |
| *Aspergillus clavatus NRRL1* | XP_001274259.1 |
| *Aspergillus flavus NRRL3357* | EED51900.1 |
| *Penicillium chrysogenum Wisconsin 541255* | XP_002565482.1 |
| *Ajellomyces dermatitidis SLH14081* | XP_002621096.1 |
| *Listeria grayi DSM 20601* | ZP_07052704.1 |
| *Listeria monocytogenes HPB2262* | ZP_05267085.1 |
| *Lactobacillus salivarius UCC118* | YP_534914.1 |
| *Lactobacillus sakei subspecies sakei 23K* | YP_394621.1 |
| *phage phiNIH1.1* | NP_438126.1 |
| *Streptococcus pyogenes NZ131* | YP_002286463.1 |
| *Streptococcus agalactiae 515* | ZP_00790494.1 |
| *Streptococcus suis 891591* | ZP_03625564.1 |
| *phage bIL286 Lactococcus lactis* | YP_001033380.1 |
| *Lactococcus lactis subsp. cremoris* | MG1363_AAK06288.1 |
| *phage bIL285 Lactococcus lactis* | NP_076587.1 |
| *phage 5093 Streptococcus* | YP_002925089.1 |
| *Enterococcus faecium E980* | ZP_06681833.1 |
| *Lactobacillus iners DSM 13335* | ZP_05743672.1 |
| *Geobacillus thermodenitrificans NG802* | YP_001127508.1 |
| *Bacillus halodurans C125* | NP_244917.1 |
| *Bacillus subtilis str. 168* | NP_391970.1 |
| *Staphylococcus aureus A9635* | ZP_05685964.1 |
| *Staphylococcus aureus subsp. aureus JKD6009* | ZP_03563033.1 |
| *phage epsilon34 Salmonella* | YP_002533495.1 |
| *phage_CP1639_Enterobacteria* | YP_002925089.1 |
| *Aeromonas_hydrophila* | YP_858391.1 |
| *Escherichia_coli* | NP_290692.1 |
| *Salmonella_enterica* | YP_001572394.1 |
| *Klebsiella_pneumoniae* | YP_001338068.1 |
| *phage_E1gi_Salmonella* | YP_001742078.1 |
| *phage_vB_EcoM_ECO123010_Escherichia_coli* | ADE87961.1 |
| *Pseudomonas_syringae* | NP_940736.1 |
| *Neisseria_gonorrhoeae_FA_1090* | YP_208119.1 |
| *Neisseria_bacilliformis_ATCC_BAA-1200* | EGF11121.1 |
| *Xanthomonas_campestris_pv._vesicatoria_str._8510* | CAJ19941.1 |
| *Tolumonas_auensis_DSM_9187* | YP_002891446.1 |
