# Supplementary material for: Characterization of the Single Stranded DNA Binding Protein SsbB Encoded in the Gonoccocal Genetic Island
Source: PLoS One. 2012 Apr 19;7(4):e35285. doi: 10.1371/journal.pone.0035285 (PMC3334931; doi:10.1371/journal.pone.0035285)
Supplement: Table S2 — Strains used in this study. (DOCX) [file pone.0035285.s004.docx]

| **Strains** | **Description** | **References** |
| --- | --- | --- |
| ***E. coli* strains** |  |  |
| DH5α | F- endA1 glnV44 thi-1 recA1 relA1 gyrA96 deoR nupG Φ80dlacZΔM15 Δ(lacZYA-argF)U169, hsdR17(rK- mK+), λ– | Invitrogen |
| Tuner (DE3) | F– ompT hsdSB(rB– mB–) gal dcm lacY1 (DE3) | Novagen |
| C43 (DE3) | F^-^ ompT hsdS_B_ (r_B_^-^ m_B_^-^) gal dcm (DE3) | (1) |
| RDP268 | F- thr-J leuB6 proA2 his4 argE3 thi-J ara-14 lacYJ galK2 xyl-5 mtl-l rpsL31 tsx-33 supE4422 ssb:Km, λ– | (2) |
| ***N. gonorrhoeae* strains** |  |  |
| MS11 | *Neisseria gonorrhoeae* strain | (3) |
| ND500 | MS11AΔGGI | (4) |
| SJ001 | MS11 strain with *pilQ* truncation | (5) |
| SJ023 | MS11 strain transformed with plasmid pSJ023. N-terminal one strep tagged *SSB*^+^*^OE^* behind a *lac* promoter inserted between *lctP* and *aspC* on the chromosome, (Cm^R^) | This study |
| SJ038 | MS11 strain transformed with plasmid pSJ038; *SSB*^+^*^OE^* behind a *lac* promoter inserted between *lctP* and *aspC* on the chromosome, (Cm^R^) | This study |
| EP006 | MS11 ΔrecA, (Erm^R^) | (6) |
| EP030 | ND500 ΔrecA, (Erm^R^) | (6) |
| EP015 | MS11 strain transformed with plasmid pKH35 vector between *lctP* and *aspC* region on the chromosome, (Cm^R^) | (6) |
| EP029 | ND500 strain transformed with plasmid pKH35 vector between *lctP* and *aspC* region on the chromosome, (Cm^R^) | (6) |

**References**

1. Miroux, B. and Walker, J.E. (1996) Over-production of proteins in Escherichia coli: mutant hosts that allow synthesis of some membrane proteins and globular proteins at high levels. *J Mol Biol*, **260**, 289-298.

2. Porter, R.D. and Black, S. (1991) The single-stranded-DNA-binding protein encoded by the Escherichia coli F factor can complement a deletion of the chromosomal ssb gene. *J Bacteriol*, **173**, 2720-2723.

3. Swanson, J., Kraus, S.J. and Gotschlich, E.C. (1971) Studies on gonococcus infection. I. Pili and zones of adhesion: their relation to gonococcal growth patterns. *J Exp Med*, **134**, 886-906.

4. Hamilton, H.L., Dominguez, N.M., Schwartz, K.J., Hackett, K.T. and Dillard, J.P. (2005) Neisseria gonorrhoeae secretes chromosomal DNA via a novel type IV secretion system. *Mol Microbiol*, **55**, 1704-1721.

5. Jain, S., Moscicka, K.B., Bos, M.P., Pachulec, E., Stuart, M.C., Keegstra, W., Boekema, E.J. and van der Does, C. Structural characterization of outer membrane components of the type IV pili system in pathogenic Neisseria. *PLoS One*, **6**, e16624.

6. Pachulec, E. and van der Does, C. (2010) Conjugative plasmids of Neisseria gonorrhoeae. *PLoS One*, **5**, e9962.
