# Supplementary material for: Characterization of the Single Stranded DNA Binding Protein SsbB Encoded in the Gonoccocal Genetic Island
Source: PLoS One. 2012 Apr 19;7(4):e35285. doi: 10.1371/journal.pone.0035285 (PMC3334931; doi:10.1371/journal.pone.0035285)
Supplement: Table S3 — Plasmids used in this study. (DOCX) [file pone.0035285.s005.docx]

| **Plasmids** | **Description** | **References** |
| --- | --- | --- |
| pET-20b(+) | Cloning/expression vector, (Amp^R^) | Novagen |
| pPR-IBA-102 | N-terminal OneStrep vector pPR-IBA-102  (Amp^R^) | IBA GmbH |
| pIDN3 | IDM vector (Erm^R^) | [[1](#_ENREF_1)] |
| pKH35 | Complementation vector (Cm^R^), 6.5kb | [[2](#_ENREF_2)] |
| pKH37 | Complementation vector (Cm^R^), 6.5kb | [[3](#_ENREF_3)] |
| pMV003 | N-terminal fusion of 10-His to *ssbB* gene cloned in the pET-20b(+) vector.  PCR product of the full length *ssbB* gene created with primers 401R-GGI and 400F-GGI on MS11A genomic DNA cloned in the NdeI and XhoI sites of pET-20b(+), (Amp) | This study |
| pMV009 | *SsbB* gene cloned in the pET-20b(+) vector.  PCR product of the full length *ssbB* gene created with primers 423F-GGI and 424R-GGI on MS11A genomic DNA cloned in the NdeI and XhoI sites of pET-20b(+), (Amp) | This study |
| pHJ002 | *ssb* gene with N-terminus one strep tag cloned in the pBR-IBA102 vector. PCR product of the full length *ssb* gene created with primers pHJ002_For and pHJ002_Rev using MS11A genomic DNA as template and cloned in the BsaI site of pBR-IBA102. | This study |
| pSJ023 | N-terminus one strep tagged *ssb* gene cloned in pKH37 vector. *ssb* gene cloned from pHJ002 in the XbaI and HindIII sites of pKH37. | This study |
| pSJ038 | *ssb* gene cloned in pKH37 vector. PCR product of the full length *ssb* gene created with primers pSJ038_For and pSJ038_Rev using MS11A genomic DNA as template and cloned in the XhoI and NdeI sites of pKH37. | This study |
| pRPZ146 | a pBR322 derivative that carries the *ssb* gene from the *E. coli* chromosome, (Tet^R^) | [[4](#_ENREF_4)] |
| pKH113 | *ssb* gene cloned in pIDN3 vector. PCR product of the full length *ssb* gene created with primers ssb-Hind and ssb-Xho using MS11A genomic DNA as template and cloned in the HindIII and XhoI sites of pIDN3. | This study |
| pKH114 | *ermC* and *ssb* gene cloned from pKH113 in pRPZ146 using PstI and SphI; replacing the *E. coli ssb* and *tet* from pRPZ146. | This study |

**References**

1. Hamilton, H.L., K.J. Schwartz, and J.P. Dillard, *Insertion-duplication mutagenesis of neisseria: use in characterization of DNA transfer genes in the gonococcal genetic island.* J Bacteriol, 2001. **183**(16): p. 4718-26.

2. Hamilton, H.L., et al., *Neisseria gonorrhoeae secretes chromosomal DNA via a novel type IV secretion system.* Mol Microbiol, 2005. **55**(6): p. 1704-21.

3. Kohler, P.L., et al., *AtlA functions as a peptidoglycan lytic transglycosylase in the Neisseria gonorrhoeae type IV secretion system.* J Bacteriol, 2007. **189**(15): p. 5421-8.

4. Porter, R.D. and S. Black, *The single-stranded-DNA-binding protein encoded by the Escherichia coli F factor can complement a deletion of the chromosomal ssb gene.* J Bacteriol, 1991. **173**(8): p. 2720-3.
