# Supplementary material for: Characterization of the Single Stranded DNA Binding Protein SsbB Encoded in the Gonoccocal Genetic Island
Source: PLoS One. 2012 Apr 19;7(4):e35285. doi: 10.1371/journal.pone.0035285 (PMC3334931; doi:10.1371/journal.pone.0035285)
Supplement: Table S4 — Primers used in this study. (DOCX) [file pone.0035285.s006.docx]

| **Primer name** | **Primer sequence** |
| --- | --- |
| pHJ002_For | 5’-GCGGTAGGTCTCAGCGCCATGTCAGTTCAACTTTTTGTTCGTG-3’ |
| pHJ002_Rev | 5’-CGCGGTAGGTCTCATATCACAATGGGATGTCATCATCAGCGT-3’ |
| pSJ038_For | 5’- GCGGCCATATGATATGTCAGTTCAACTTTTTGT  TCGTGG-3’ |
| pSJ038_Rev | 5’- GCGCCTCGAGCGTGGCCATATATTTACAATGGG-3’ |
| ssb-Hind | 5’-GCTAAGCTTTCAGCCCATAATGCAGCAAG-3’ |
| ssb-Xho | 5’-ACTCGAGATGACTGTCCGTGGGCATTT-3’ |
| 400F-GGI | 5’-CACACCTCGAGTTACAATGGGATGTCATCATCAGC-3’ |
| 401R-GGI | 5’-CTCTCCATATGCACCATCACCATCACCATCACCATCACCACATGTCAGTTCAACTTTTTGTTCG-3’ |
| 407F-GGI | 5’-AGGGAAGGTCTCGTATCATTACAATGGGATGTCATCATCAGC-3’ |
| 423F-GGI | 5’-AGGCCACTCGAGTTACAATGGGATGTCATCATCAGC-3’ |
| 424R-GGI | 5’-AGGCCACATATGTCAGTTCAACTTTTTGTTCG-3’ |
| 427R-GGI | 5’-GTACGATTGAGGCTTCACAGTTTTTAGGGCTAGCTACAGGACGC-3’ |
| 428F-GGI | 5’-TGAAGCCTCAATCGTACTGAATTTTTCAGTTGCCTCACC-3’ |
| 472R-GGI | 5’-GATATGCCCGAGTCTGAAGC-3’ |
| 473F-GGI | 5’-CCCAATGCGTCAATAAGAGG-3’ |
| 474R-GGI | 5’-GTCTATCCAACCGGTGACAG-3’ |
| 475F-GGI | 5’-CCCGGTTCTTTAGCTTTCTC-3’ |
| 498R-GGI | 5’-GACGGAATGCGACTATTGAG-3’ |
| 499F-GGI | 5’-ACTTTCCAGTATGCTGGTAGAGGGC-3’ |
| 701R-GGI | 5’-GTGGTTGAACACCGACAATC-3’ |
| 702F-GGI | 5’-TTACGGTAGCCACAGTAGTC-3’ |
| 703R-GGI | 5’-CTGAGCGTGTAGAAGCTATC-3’ |
| 705R-GGI | 5’-TTCTGTGCCGATGACTGTCC-3’ |
| 708R-GGI | 5’-ATGTCTGTCCGACCTGTAAG-3’ |
| 709F-GGI | 5’-CTTCAGGATTGTCGGTGTTC-3’ |
| 726F-GGI | 5’-GGTTAAGTTGCGGCTTTCAC-3’ |
| 766R-GGI | 5’-ATCGTGATGCTGCCCATCTC-3’ |
| 767F-GGI | 5’-ACGCTCAGTTGGAACAATGAATAC-3’ |
| 769F-GGI | 5’-CCTGCCACAGTGTAGTAAAC-3’ |
| 770R-GGI | 5’-TCGATCGGACGGATTCAAAC-3’ |
| 697 | 5’-GCTTACGGCGTTGCTTATTG-3’ |
| 698 | 5’-CCCGCCCTACCATTAAACTG-3’ |
