# Supplementary material for: Characterization of the Single Stranded DNA Binding Protein SsbB Encoded in the Gonoccocal Genetic Island
Source: PLoS One. 2012 Apr 19;7(4):e35285. doi: 10.1371/journal.pone.0035285 (PMC3334931; doi:10.1371/journal.pone.0035285)
Supplement: Table S5 — Organization of the genetic cluster surrounding N. gonorrhoeae SsbB. (DOCX) [file pone.0035285.s007.docx]

| **Gene name** | **protein length (aa)** | **protein function** | **distance between stop codon and the start codon of the adjacent gene (bp)** |
| --- | --- | --- | --- |
| *yfb* | 349 | conserved hypothetical protein with a DUF1845 domain | 115 |
| *yfa* | 184 | hypothetical protein, no homology | 586 |
| *ssbB* | 143 | single stranded DNA binding protein | 190 |
| *topB* | 679 | Topoisomerase I | 13 |
| *yeh* | 188 | hypothetical protein, no homology | 11 |
| *yegB* | 32 | hypothetical protein, no homology | overlap of 7 |
| *yegA* | 190 | Belongs to Peptidase_M15_2 family (DUF882 ) with conserved hypothetical proteins of unknown function | 262 |
| *yef* | 149 | conserved hypothetic protein with a DUF3577 domain | 19 |
